# Huaier extract suppresses breast cancer via regulating tumor-associated macrophages

**DOI:** 10.1038/srep20049

**Published:** 2016-02-01

**Authors:** Yaming Li, Wenwen Qi, Xiaojin Song, Shangge Lv, Hanwen Zhang, Qifeng Yang

**Affiliations:** 1 Department of Breast Surgery, Qilu Hospital, Shandong University, School of Medicine, Wenhua West Road No. 107, Ji’nan, Shandong 250012, P. R. China; 2 Department of Radiation Oncology, UMDNJ-Robert Wood Johnson School of Medicine, and the Cancer Institute of New Jersey, New Brunswick, NJ, U.S.A

## Abstract

Macrophages in tumor microenvironment are mostly M2-polarized - and have been reported to promote tumorigenesis, which are also defined as tumor-associated macrophages (TAMs). Here, we examined the regulatory effects of Huaier extract on TAMs using RAW264.7 murine macrophage cell line. Our data demonstrated that Huaier extract could inhibit the infiltration of macrophages into tumor microenvironment in a dose-dependent manner. By performing RT-PCR, immunofluorescence and phagocytosis assay, we were able to find that Huaier extract could regulate the polarization of macrophages, with decreased M2-polarization and increased phagocytosis of RAW264.7 cells. Moreover, we identified that Huaier extract could suppress macrophages-induced angiogenesis by using HUVEC migration assay, tube formation and chorioallantoic membrane assay. Additionally, western blotting showed decreased expression of MMP2, MMP9 and VEGF with the use of Huaier extract. Finally, we found that Huaier extract could inhibit M2-macrophages infiltration and angiogenesis through treating 4T1 tumor bearing mice with Huaier extract. Our study revealed a novel mechanism of the anti-tumor effect of Huaier extract which inhibited angiogenesis by targeting TAMs. These findings provided that Huaier was a promising drug for clinical treatment of breast cancer.

The tumor microenvironment is a complex system composed of many cell types including endothelial cells, smooth muscle cells, fibroblasts, and inflammatory cells such as macrophages and dendritic cells. It is a unique environment which emerges in the course of tumor progression as a result of its interaction- with the host^1^. Macrophages are the major inflammatory components of the stroma which shift their functional phenotypes in response to various microenvironmental signals generated from tumor and stromal cells. Therefore, they are defined as tumor-associated macrophages (TAMs)^2^. Although under certain circumstances macrophages can kill tumor cells, they can also act as tumor promoters by secreting a variety of factors that directly stimulate tumor growth, metastasis and angiogenesis^3^. Macrophages in tumor microenvironment are divided into two distinct phenotypes: the classically activated (M1) phenotype, which is involved in the inflammatory response, pathogen clearance and antitumor immunity; and the alternatively activated (M2) phenotype, which has anti-inflammatory, pro-angiogenic and pro-tumoral properties^4^. Within the tumor microenvironment, TAM-induced angiogenesis is associated with cancer progression and proliferation^5^. Recently, accumulating evidences have demonstrated that macrophages promote angiogenesis in tumors by producing excessive amounts of pro-angiogenic factors and by physically assisting sprouting blood vessels to augment the complexity of the intra-tumoral vascular network^6^. Given that, we speculated that specifically targeting TAMs or reprogramming them from a pro-angiogenic to an angiostatic function may “normalize” the tumor vasculature and improve the efficacy of various anticancer therapies.

Recently, much attention has been focused on medicinal plants or herbs as potential sources of new therapeutic anticancer drugs due to their lack of toxic effects, relatively lower price, and more effectiveness, among which traditional Chinese medicine (TCM) has gained growing popularity for its novel role in killing tumor cells less intensively and more naturally^7^,^8^. Trametes robiniophila Murr (Huaier) is a sandy beige mushroom found on the truck of trees and has been widely used in TCM for approximate 1600 years. However, its antitumor properties were found and used as a complementary therapy only in recent decades. Previous studies have reported that Huaier could inhibit the growth, metastasis, angiogenesis of tumor and exert immune-modulatory effects^9^,^10^,^11^. These data indicated that Huaier may serve as a novel potent anticancer agent of broad clinical value. Nevertheless, it remains unknown whether Huaier could inhibit breast cancer by target TAMs. In the present study, we evaluated the regulatory effects of Huaier on TAMs, in combination with its antitumor effects.

## Materials and Methods

### Materials

Dulbecco’s Modified Eagle’s Medium (DMEM) was purchased from Gibco-BRL (Rockville, IN, USA). Fetal bovine serum (FBS) was supplied by Haoyang Biological Manufacturer Co., Ltd (Tianjin, China). Anti-MMP2, anti-MMP9 antibodies were bought from Cell Signaling Technology (Beverly, MA, USA). Anti-VEGF, anti-CD206 antibodies were obtained from Abcam (Cambridge, MA, USA). Anti-CD31 antibody was provided by Immunoway Biotechnology Company (Newark, DE, USA). Anti-mouse IgG horseradish peroxidase (HRP) antibody was from ZhongShan Goldenbridge (Beijing, China). Fluorescein isothiocyanate–dextran and LPS were purchased from Sigma–Aldrich (St Louis, MO, USA). Murine INF-γ, IL-4 and IL-13 were bought from Peprotech (Rocky Hill, NJ, USA). The study was approved by the institutional guidelines of the Animal Care and Use Committee at Shandong University. All the methods used in our study were carried out in accordance with the approved guidelines.

### Cell culture

All cell lines were purchased from American Type Culture Collection (ATCC), and were routinely cultured in DMEM medium supplemented with 10% FBS, 100 U⁄mL penicillin and 100 μg⁄mL streptomycin under the conditions of 5% CO_2_ at 37 °C.

### Preparation of Huaier aqueous extract

Electuary ointment of Huaier extract was kindly donated by Gaitianli Medicine Co. Ltd (Jiangsu, China). Two grams of electuary ointment was dissolved in 20 mL DMEM and was filtered through a 0.22-μm filter to make the 100 mg/mL solution for long term storage at −20 °C 12. The components of Huaier was provided in supplementary information as Table S1. **Preparation of conditioned media.** RAW264.7 and 4T1 cells were treated with different concentrations of Huaier extract for 48 h in 100-mm culture discs. The cells were washed with phosphate-buffered saline (PBS) for three times and cultured in DMEM for another 24 h. The culture supernatants (conditioned media, CM) were collected and centrifuged to remove cellular components and stored in 4 °C.

### Transwell migration assay

*In vitro* cell migration assay was performed using the Transwell system (24-wells, 8-μm pore size with polycarbonate membrane; Corning Costar, Lowell, MA, USA). Briefly, RAW264.7 cells and HUVEC cells were harvested and suspended in serum-free medium, then 1 × 10^5^ cells were added to the upper wells. The conditioned media from 4T1 and RAW264.7 cells treated with different concentrations of Huaier extract were mixed with complete media (v/v 1:1), then 700 μl of the mixture were added to the lower chamber. After treatment for 24 h, the cells attached to the lower surface were fixed with methanol and stained with 0.2% Giemsa. The number of cells migrated were acquired in five randomized fields using an Olympus light microscope^13^.

### Immunofluorescence microscopy

Cells grown on glass coverslips were rinsed once with PBS and fixed in paraformaldehyde for 20 min at room temperature (RT). Cells were then washed 3 times with PBS and blocked with 10% goat serum for 45 min. Cells were then incubated with rabbit anti-mouse CD206 (1:100) overnight in 4 °C, washed again, and incubated with Rhodamine-conjugated goat anti-rabbit IgG for 1 h. After washing with PBS, cells were further stained with 4, 6-diamidino-2-pheny-lindole (DAPI) for 5 min. Finally, cells were washed, mounted, and examined with an Olympus light microscope.

### Phagocytosis assay

To quantify the phagocytosis ability of - Huaier-treated macrophages, FITC–Dextran uptake was monitored by FACS as described by Stumbles *et al.*^14^. Briefly, 2 × 10^5^ macrophages were suspended in 100 μl DMEM, then 50 μl 4 mg/ml dextran (molecular weight 40,000) was added for 1 h at 37 °C or 4 °C. Cells were washed three times with PBS and the fluorescence intensity was determined by FACS.

### Tube formation assay

The ability of HUVEC cells given to different conditioned media from RAW264.7 cells to form network structures was tested on Matrigel basement membrane matrix (BD Biosciences, San Jose, CA, USA). First of all, 70 μl Matrigel were plated per well on 96-well plates and incubated for 30 min. Subsequently, 100 μl of HUVEC cells suspended in RAW264.7-CM containing 5% FBS at a density of 1.5 × 10^5^/ml were added to each plates. After 4 h of incubation at 37 °C, tube-like structures were photographed with an Olympus digital camera.

### Chick chorioallantoic membrane (CAM) assay

The angiogenic activity was evaluated using a CAM assay as described previously^15^ with small modifications. Briefly, fertilized chicken eggs (6 eggs/group) were incubated at 38 °C in an 80% humidified atmosphere. On developmental day 7, a square window was opened in the shell. On the following day, gelatin sponges (0.3 cm × 0.3 cm × 0.3 cm) containing PBS or RAW264.7-CM were placed on the top of the growing CAMs under sterile conditions. After 72 h of incubation, the CAMs were photographed using an Olympus Live View Digital SLR camera.

### Animals and tumor model

Twelve BALB/c female mice, 4–5 weeks old, purchased from the Center for New Drugs Evaluation of Shandong University, were randomly divided into two groups. All the experiments were approved by the institutional guidelines of the Animal Care and Use Committee at Shandong University. 4T1 cells (1 × 10^6^) were subcutaneously injected into the left hypogastrium of each mouse. After 2 days, 150 μl solution containing 75 mg Huaier extract or water was given by gavage to each mouse every day as therapeutic models. After 21 days, the mice were sacrificed, and the tumors were removed for immunohistochemical staining.

### Immunohistochemistry (IHC)

The tumor tissues were fixed in 10% neutral-buffered formalin. After 48 h, the tissues were paraffin-embedded and then sliced into 4-μm section for further IHC. Briefly, the sections were deparaffinized and rehydrated, followed by antigen retrieval with pH 6.0 citrate buffer. 3% H_2_O_2_ was used to inhibit endogenous peroxidase activity, and the sections were incubated with 10% normal goat serum to block non-specific binding. After incubation with anti-CD31 antibody or anti-CD206 antibody at 4 °C overnight, the sections were washed with PBS and then treated with biotinylated anti-immunoglobulin antibody for 30 min and reacted with HRP-conjugated streptavidin. Then the DAB substrate system (Maixin Bio, Fuzhou, China) was used, followed by counterstaining with hematoxylin. The images of tumor tissues were taken by an Olympus light microscope. The number of TAMs in each field was automatically counted using Image-pro plus 6.0, and the MVD was manually assessed at ×100 magnification simultaneously by two experienced investigators.

### Real-time PCR

Total RNA was isolated with TRIZOL reagents according to the manufacturer’s protocol. cDNA was synthesized from 1 μl of total RNA by a PrimerScript RT Reagent kit (TaKaRa). Real-time quantitative RT-PCR was performed using a SYBR green PCR mix in Applied Biosystems 7900HT Real-Time PCR System. The mRNA expression from each sample was calculated by normalizing with endogenous control GAPDH. The experiments were repeated in triplicate to confirm the findings. The primers used in our study were provided as Table S2 in supplementary information.

### Western blot analysis

The RAW264.7 cells treated with different concentrations of Huaier extract were washed twice with cold PBS and were lysed with lysis buffer on ice. The protein concentration was determined using a BCA protein concentration kit. 80 μg of each samples were resolved in SDS-PAGE gels and then transferred onto a PVDF membrane (Millipore, Bedford, MA, USA). The membrane was blocked with 5% nonfat milk for 1 h at RT and then incubated with primary antibody overnight at 4 °C. After washing with PBST, the membrane was incubated with the secondary antibody conjugated with HRP and the proteins were detected by a chemiluminescence system (ECL kit).

### Statistical analysis

Data are presented as the mean ± standard error of the mean (SEM). Statistical comparisons between 2 samples were performed using the Student’s t-test. A P-value less than 0.05 was considered statistically significant. The SPSS version 18.0 software was used for statistical analysis.

## Results

### Huaier extract decreases M2 macrophage infiltration into the breast cancer microenvironment

**Figure 1:**
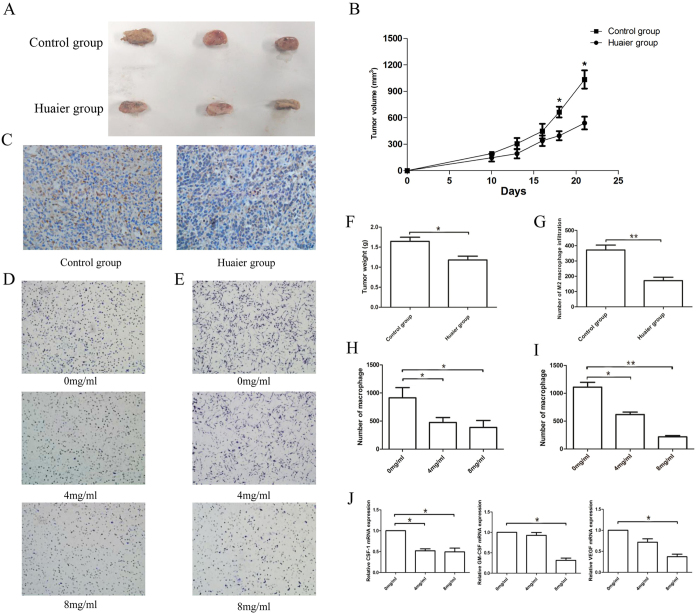
Huaier extract inhibited the proliferation of 4T1 cells and infiltration of macrophages. (**A**) Huaier extract inhibited 4T1 cells growth *in vivo* after administrated with Huaier extract for 21 days. (**B**) The tumor growth curves. (**C**) Representative IHC images of CD206 positive macrophages in tumor tissues. (**D**) 24 h migration assay of RAW264.7 cells treated with 0 mg/ml, 4 mg/ml, 8 mg/ml Huaier extract. (**E**) 24 h migration assay of RAW264.7 cells to CM from 0 mg/ml, 4 mg/ml and 8 mg/ml treated 4T1 cells. (**F**) The tumor weight in control group and Huaier group. (**G**) The number of M2 positive macrophages infiltration in control group and Huaier group. (**H**) The number of macrophages migrated in Figure C. (**I**) The number of macrophages migrated in Figure D. (J) The relative mRNA expression of CSF-1, GM-CSF and VEGF. Results are presented as the mean ± SEM of triplicate samples. *P < 0.05, **P < 0.01.

It has been reported that Huaier exhibited anti-tumor and immunomodulatory effects^9^. We observed that the proliferation and size of tumors in the Huaier group was significantly reduced compared to control group (Fig. 1A,B,F), suggesting that Huaier could inhibit the growth of tumors in syngeneic models.

Previous studies have shown that M2-polarized macrophages infiltration plays a critical role in the tumorigenic and invasive progress, which could be a potential target of anti-cancer therapy^16^. To test whether Huaier extract could also inhibit tumor progression via the inhibition of M2 macrophages infiltration in tumor microenvironment, we first tested the influence of Huaier extract on macrophage motility. RAW264.7 cells were pre-treated with 0, 4, 8 mg/ml Huaier extract for 48 h. Migration assay showed that Huaier treatment could significantly inhibit the motility of RAW264.7 cells in a dose dependent manner (Fig. 1D,H). We also examined the RAW264.7 cell migration response to conditioned medium from different concentration (0 mg/ml, 4 mg/ml, 8 mg/ml) treated breast cancer 4T1 cells. We found that the migration of RAW264.7 cells was greatly decreased in the conditioned medium from Huaier-treated 4T1 cells in a dose-dependent manner (Fig. 1E,I). To understand the mechanism, 4T1 cells were treated with Huaier extract for 48 h, and 4T1 cell-secreted CSF-1, GM-CSF and VEGF were examined for their chemotactic effect on macrophages (Fig. 1J)^17^. Our data demonstrated that Huaier extract could not only decrease the motility by directly act on macrophages, but also inhibit the tumor-induced chemotaxis of macrophages. To further examine the number of M2-positive macrophages *in vivo*, immunohistochemical assay against CD206, which is the marker of M2 macrophages, was performed. As shown in Fig. 1C,G, number of CD206-positive macrophages in the tumor was significantly reduced after treatment with Huaier. In summary, these results showed that Huaier extract could decrease macrophage infiltration in breast cancer microenvironment.

### Huaier extract inhibits M2 macrophages polarization and promotes macrophages phagocytosis

**Figure 2:**
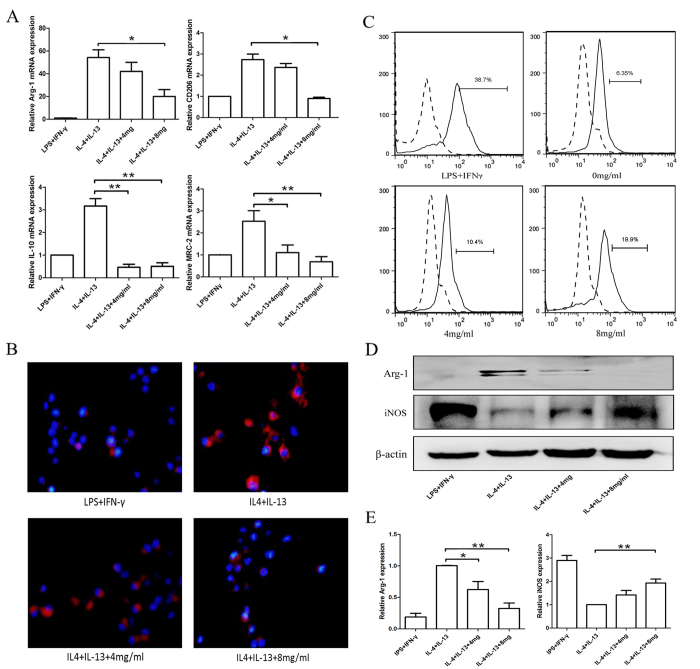
Huaier reduced M2 macrophages polarization and enhanced phagocytosis of RAW264.7 cells. (**A**) RAW264.7 cells were treated with LPS (100 ng/ml) + IFN-γ(20 ng/ml) for 24 h as negative control, and treated with IL-4 (20 ng/ml) + IL-13 (20 ng/ml) +0, 4, 8 mg/ml Huaier extract for 24 h. The relative mRNA expression of M2 macrophages markers including Arg-1, CD206, IL-10 and MRC-2 was examined using qPCR. (**B**) Immunofluorescence of CD206 after treatment with LPS + IFN-γ, IL-4 + IL-13, IL-4 + IL-13 + 4 mg/ml, or IL-4 + IL-13 + 8 mg/ml Huaier extract for 24 h. (**C**) FITC-Dextran phagocytosis assay of RAW264.7 cells after treated with Huaier extract for 24 h. Cells treated with LPS + IFN-γ was used as a positive control. (**D**) Western blotting of Arg-1 and iNOS protein expression. (**E**) Relative Arg-1 and iNOS protein expression level. Results are presented as the mean ± SEM of triplicate samples. *P < 0.05, **P < 0.01.

TAMs are often characterized as displaying an M2 phenotype^4^, which could facilitate the tumor progression and are negatively correlated with patients prognosis in breast cancer^2^. Previous studies have shown that naïve macrophages could be quickly polarized to M2 phenotype in tumor microenvironment^18^. To determine whether Huaier extract could inhibit the M2 polarization of RAW264.7 cells, several previously identified M2 macrophage markers were used, including CD206, mannose receptor C-2 (Mrc-2), arginase-1 (Arg-1), and IL-10^19^,^20^,^21^. The naïve RAW264.7 macrophages were treated with LPS + IFN-γ(negative control), IL-4 + IL-13 (positive control) or IL-4 + IL-13 + 4, 8 mg/ml Huaier extract for 48 h. As shown in Fig. 2A, compared with the positive control, the mRNA expression level of the M2 macrophages markers was significantly reduced after treating with Huaier extract. Because CD206 is the M2 phenotype marker, we further examined the expression of CD206 using immunofluorescence. Consistent with our previous results, the fluorescence intensity was significantly decreased in a dose-dependent manner (Fig. 2B). Taken together, these results proved that Huaier extract could inhibit the polarization of innate RAW264.7 cells to an M2 phenotype, suggesting that Huaier could alter proinflammatory cytokine expressed by the macrophages, and thus could promote the immune response within the tumor microenvironment.

Phagocytosis of antigens, pathogens and dying cells is one of the most important roles of macrophages. To assess whether pre-treated with Huaier could alter the phagocytic activity, FITC–Dextran was used as a model antigen and its uptake was measured by FACS. RAW264.7 cells were pretreated with LPS + IFN-γ(positive control) and Huaier extract(0 mg/ml, 4 mg/ml, 8 mg/ml). As shown in Fig. 2C, pre-treated with Huaier extract could remarkably enhance the uptake of dextran by RAW264.7 macrophages in a dose-dependent manner. Finally, we determined the protein levels of Arg-1 and iNOS in M2 macrophages treated with different concentrations of Huaier extract. As shown in Fig. 2D,E, the expression of M2 marker Arg-1 was significantly reduced after treated with Huaier extract while the M1 marker iNOS was increased. Our results demonstrated that Huaier extract could regulate the polarization of macrophages.

### Huaier extract inhibits RAW264.7 induced angiogenesis and decreases MMP2/MMP9/VEGF expression

**Figure 3:**
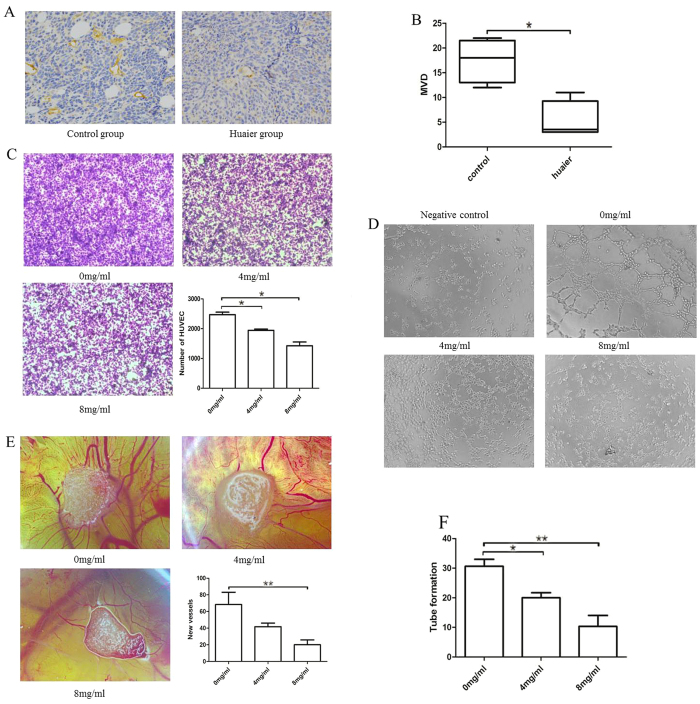
Huaier extract inhibited the macrophages induced angiogenesis. (**A**) Representative IHC images of CD31 in tumor tissues in Huaier group and control group. (**B**) Micro vessels density in control group and Huaier group. (**C**) HUVEC migration response to CM from 0 mg/ml, 4 mg/ml, 8 mg/ml Huaier extract treated RAW264.7 cells for 24 h. (**D**) Tube formation assay of HUVEC cells treated with DMEM + 5%FBS (Negative control) or CM + 5%FBS for 4 h. (**E**) CAM assay treated with CM from 0 mg/ml, 4 mg/ml, 8 mg/ml Huaier extract treated RAW264.7 cells for 72 h. (**F**) Number of complete tube-like structures. Results are presented as the mean ± SEM of triplicate samples. *P < 0.05, **P < 0.01.

It is well known that forming new blood vessels is an essential step in tumor development. Without blood supply, the tumor volume will not exceed 1-2 mm^3^ 22,23. We first verified the anti-angiogenic effect of huaier extract *in vivo* using IHC to anti-CD31 antibody. As shown in Fig. 3A,B, number of new blood vessels in Huaier group was remarkably reduced compared with the control group. Because macrophages play a critical role in the angiogenesis progress of the tumor^17^, we explored whether the anti-angiogenic effect of Huaier extract was partly induced by targeting macrophages. RAW264.7 cells were treated with Huaier extract (0 mg/ml, 4 mg/ml and 8 mg/ml) for 48 h and conditioned medium (RAW264.7-CM) were collected. Migration assay using HUVEC and RAW264.7-CM showed a decreased chemotactic effect of Huaier-treated macrophages on vascular endothelial cells (Fig. 3C). As an essential step for angiogenesis, the formation of tube-like structures involves in matrix degradation, rearrangement and apoptosis of endothelial cells^10^. As shown in Fig. 3D,F, number of complete network structures was significantly decreased in a dose-dependent manner, showing that Huaier extract could inhibit macrophages induced formation of vascular structure. To verify the effect of Huaier extract on the macrophages induced angiogenesis *ex vivo*, we further performed CAM assay. After incubation in 38 °C for 72 h with different RAW264.7-CM, new vessels were remarkable reduced in a dose-dependent manner, with the CM from 8 mg/ml-treated RAW264.7 cells showing the minimal formation of new vessels (Fig. 3E).

**Figure 4:**
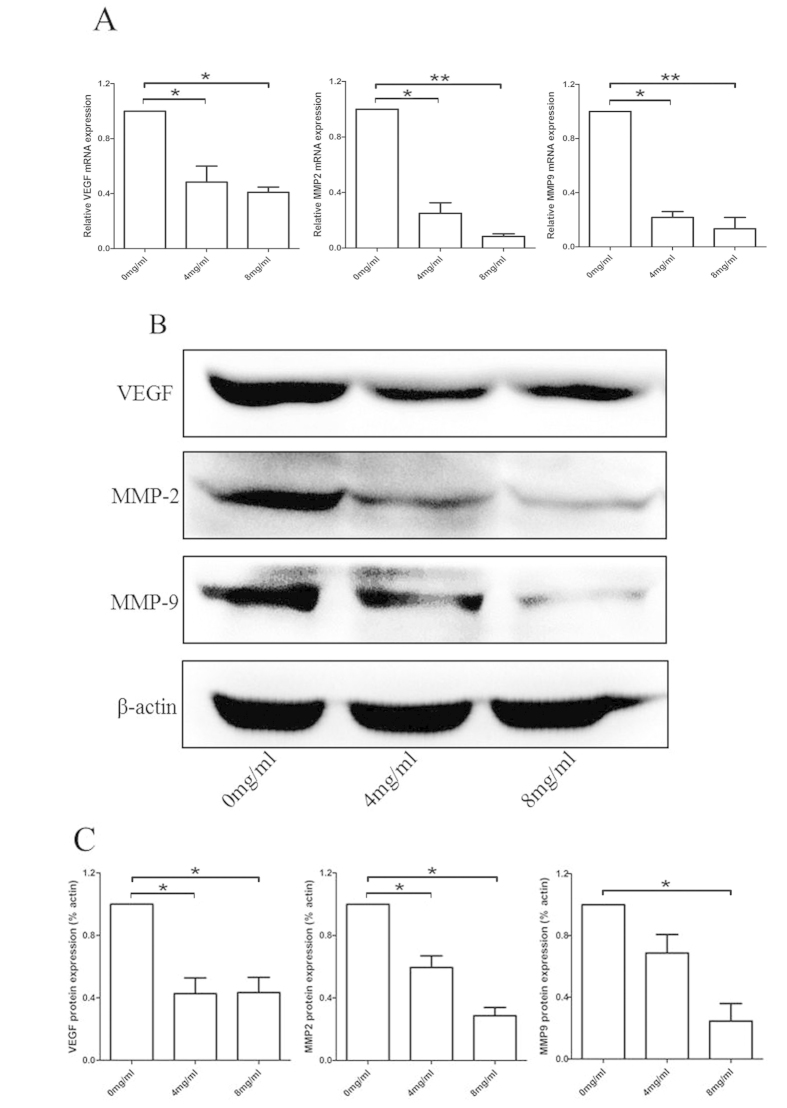
The expression of angiogenesis associated cytokines in Huaier treated RAW264.7 cells. (**A**) Relative VEGF, MMP2, MMP9 mRNA expression. (**B**) Western blotting of VEGF, MMP2 and MMP9 protein expression. (**C**) Relative VEGF, MMP2, MMP9 protein expression level. Results are presented as the mean ± SEM of triplicate samples. *P < 0.05, **P < 0.01.

Matrix metalloproteinases (MMPs) are proteolytic enzymes that remodel the extra-cellular matrix as part of an inflammatory response. One of the major MMP species in the vasculature is the gelatinases (MMP2 and MMP9), which can regulate vascular matrix remodeling^24^. Due to the important role of MMP9, MMP2 and VEGF in the progress of angiogenesis, we further examined the expression of MMP9, MMP2 and VEGF using RT-PCR and western blotting. As shown in Fig. 4A–C, after treating with Huaier extract for 48 h, the expression of MMP9, MMP2 and VEGF was significantly reduced both in mRNA and protein levels in a dose-dependent manner. In summary, these results demonstrated that Huaier extract could inhibit the macrophages induced angiogenesis by decreasing expression of VEGF, MMP2 and MMP9, and thus inhibit the formation of new blood vessels in tumor.

## Discussion

Breast cancer is the leading malignant tumor among women. Conventional treatments include surgery, radiotherapy and chemotherapy^25^. However, most of the treatments cause irreversibly damage to patients. Recently, TCM has become a rich source for finding new drugs due to its novel role in killing tumor cells less intensively and more naturally, reducing toxic side-effects, improving quality of life, enhancing immune function as well as preventing recurrence and metastasis for cancer patients^7^,^8^. One of the TCMs, Trametes robiniophila murr (Huaier) has been used in China for more than 1600 years, whereas its anti-tumor and immunomodulatory effects have only been studied in the past few years^9^,^26^. Based on its immunomodulatory and anti-tumor effects, we assessed the effect of Huaier extract on the regulation of macrophages in tumor microenvironment using RAW264.7 murine macrophage cells.

TAMs refers to macrophages in the tumor microenvironment which come from monocytes and can be attracted to the tumor tissue by chemotactic factors including VEGF, CSF-1, GM-CSF and so on^27^. Previous studies have shown that macrophages in tumor tissue predict a bad prognosis in patients^2^,^28^. Qian *et al.* have reported that macrophages infiltration could lower the prognosis of patients in breast cancer, colon cancer, prostatic cancer and esophagus cancer^2^. In the present study, we explored whether Huaier could decrease the infiltration of M2-macrophages *in vitro* and *in vivo*. Transwell migration assay confirmed that with the Huaier extract, the number of RAW264.7 macrophages cells migrated was remarkably decreased in a dose-dependent manner, showing that Huaier extract could inhibit the M2-macrophages infiltration by targeting macrophages themselves or tumor cells (Fig. 1D,E,H,I). The mRNA levels of CSF-1, GM-CSF and VEGF also verified our results (Fig. 1J). The number of M2-macrophages infiltration was also tested *in vivo*, as shown in Fig. 1C,G. The number of macrophages was significantly reduced in the group treated with Huaier extract. In summary, the results proved our hypothesis that Huaier extract could inhibit the M2-macrophages infiltration.

Macrophages in tumor tissue were polarized to two phenotypes including fewer M1 and more M2 macrophages. The M1-polarized macrophages release cytokines such as TNF-α and IL-12, and have an anti-tumor effect; The M2-polarized macrophages express high levels of IL-10, Arg-1, CD206, VEGF, and EGF, which could promote the proliferation, metastasis, and angiogenesis of tumors^4^,^5^,^29^,^30^. Previous studies have shown that infiltration of M2-macrophages is negatively related to patients’ prognosis for their modulation of the tumor microenvironment by attenuating the anti-tumor immune response, remodeling the ECM, and enhancing angiogenesis^2^,^28^,^31^. In the present study, we demonstrated that Huaier could regulate the polarization of macrophages, which could inhibit M2-phenotype polarization and decrease the expression of M2-phenotype markers and increase the expression of M1-phenotype marker (Fig. 2A,B,D,E). Phagocytosis is the most important role of macrophages, as many studies have proved that increased phagocytic activity could inhibit the proliferation of tumor cells^32^,^33^. We proved that after treating with Huaier extract, the phagocytic activity of RAW264.7 cells was significantly increased in a dose-dependent manner (Fig. 2C). Taken together, we demonstrated that Huaier could regulate the polarization and phagocytic effect of macrophages and thus have an anti-tumor effect.

Angiogenesis is an indispensable step for the growth and metastasis of tumors and thus becomes a promising target in anticancer therapy. TAMs have been shown to play a critical role in modulating angiogenesis in the tumor environment and can release many pro-angiogenic factors such as MMP2/9 and VEGF^5^,^17^,^34^. Huaier has been reported to inhibit angiogenesis by directly targeting of vascular endothelial cells, but no report has been focused on the macrophages^10^. As shown in Fig. 3A,B, we confirmed that Huaier could inhibit angiogenesis *in vivo*. Figure 3C showed a decreased chemotactic effect of RAW264.7-CM to vascular endothelial cells after treated with Huaier extract. Studies examined the effect of Huaier extract on the ability of forming tube-like structures (Fig. 3D,F) and new blood vessels (Fig. 3E) confirmed that Huaier could inhibit the macrophages-induced angiogenesis. As shown in Fig. 4, RT-PCR and western blotting showed that Huaier could decrease the expression of MMP2, MMP9 and VEGF in both mRNA and protein levels, which confirmed that Huaier could inhibit the macrophages-induced angiogenesis.

## Conclusion

**Figure 5:**
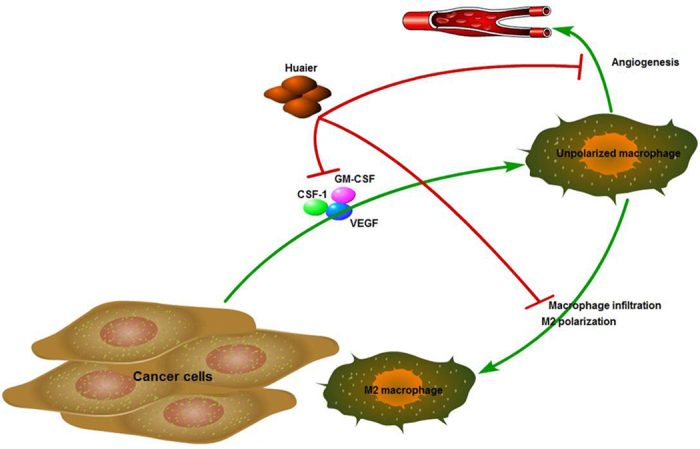
Huaier extract inhibited macrophages infiltration to tumor microenvironment and 540 polarization to M2 phenotype, increased the phagocytosis, and decreased the 541 macrophages-induced angiogenesis. The figure was drawn by L.Y.

In conclusion, Huaier is a potent anti-tumor and immunomodulatory drug, which could inhibit the infiltration of M2-macrophages in breast cancer microenvironment, regulate the polarization of TAMs, increase the phagocytosis, and decrease the angiogenesis of macrophages (Fig. 5). These results suggested a new mechanism of Huaier extract on the inhibition of breast cancer cells and contributed to the application of Huaier on the clinical treatment of breast cancer patients. Further studies are needed to assess the detailed mechanisms and pathways to better understand the anti-tumor effect of Huaier.

## Additional Information

**How to cite this article**: Li, Y. *et al.* Huaier extract suppresses breast cancer via regulating tumor-associated macrophages. *Sci. Rep.* **6**, 20049; doi: 10.1038/srep20049 (2016).

## Supplementary Material

Supplementary Information
