# Supplementary material for: Huaier extract suppresses breast cancer via regulating tumor-associated macrophages
Source: Sci Rep. 2016 Feb 1;6:20049. doi: 10.1038/srep20049 (PMC4735520; doi:10.1038/srep20049)
Supplement: Supplementary Information [file srep20049-s1.doc]

Huaier extract suppresses breast cancer via regulating tumor-associated macrophages

Li Yaming^1^, Qi Wenwen^1^, Song Xiaojin^1^, Lv Shangge ^1^, Zhang Hanwen^1^, Yang Qifeng^1, 2*^

^1^Department of Breast Surgery, Qilu Hospital, Shandong University, School of Medicine, Wenhua West Road No. 107, Ji'nan, Shandong 250012, P. R. China

^2^Department of Radiation Oncology, UMDNJ-Robert Wood Johnson School of Medicine, and the Cancer Institute of New Jersey, New Brunswick, NJ, U.S.A.

^*^Corresponding author. Prof. Qifeng Yang, at Department of Breast Surgery, Qilu Hospital, Shandong University, School of Medicine, China. E-mail address: qifengyang_sdu@126.com, or Prof. Bruce G. Haffty, at Department of Radiation Oncology, UMDNJ-Robert Wood Johnson School of Medicine, and the Cancer Institute of New Jersey, New Brunswick, NJ, U.S.A.

Table S1. The chemical composition of proteoglycan extracted from Huaier

| Composition of Amino acid^1^ | | | |  | Composition of polysaccharide^2^ | |
| --- | --- | --- | --- | --- | --- | --- |
| A.A. | percent | A.A. | percent |  | monosaccharide | molar ratio |
| Asp | 1.418 | Met | 0.120 |  | L-Fucose | 0.51 |
| Thr | 0.731 | Ile | 0.398 |  | L-Arabinose | 1.15 |
| Ser | 0.626 | Leu | 0.569 |  | D-xylose | 1.48 |
| Glu | 3.525 | Tyr | 0.249 |  | D-mannos | 1.39 |
| Pro | 0.740 | Phe | 0.347 |  | D-galactos | 1 |
| Gly | 1.073 | Lys | 0.689 |  | D-Glucose | 3.24 |
| Ala | 0.6624 | His | 0.267 |  |  |  |
| Cys | 0.160 | Try | 0.097 |  |  |  |
| Val | 0.652 | Arg | 0.645 |  |  |  |

[1] Guo Y, Cheng P, Chen Y, et al. Isolation and analysis of the polysaccharide of Huaier mycelium. Chinese J Biochem Pharm. 1993; 63: 56-9.

[2] Guo Y, Cheng P, Chen Y, et al. Studies on the Constituents of Polysaccharide from the Hyphae of Trametes Robiniophila(II)——Identification of Polysaccharide from the Hyphae of Trametes Robiniophila and Determination of Its Molar Ratio. J Chinese Pharm U. 1992; 23: 155-7.

Table S2. Primers used in the study

| CSF-1 F primer | GCCTCCTGTTCTACAAGTGGAAG |
| --- | --- |
| CSF-1 R primer | ACTGGCAGTTCCACCTGTCTGT |
| GM-CSF F primer | AACCTCCTGGATGACATGCCTG |
| GM-CSF R primer | AAATTGCCCCGTAGACCCTGCT |
| VEGF F primer | CTGCTGTAACGATGAAGCCCTG |
| VEGF R primer | GCTGTAGGAAGCTCATCTCTCC |
| Mrc-2 F primer | ATCCAGGGAAACTCACACGGA |
| Mrc-2 R primer | GCGCTCATCTTTGCCGTAGT |
| ARG-1 F primer | CATTGGCTTGCGAGACGTAGAC |
| ARG-1 R primer | GCTGAAGGTCTCTTCCATCACC |
| IL-10 F primer | CGGGAAGACAATAACTGCACCC |
| IL-10 R primer | CGGTTAGCAGTATGTTGTCCAGC |
| CD206 F primer | CTCTGTTCAGCTATTGGACGC |
| CD206 R primer | CGGAATTTCTGGGATTCAGCTTC |
| MMP2 F primer | CAAGGATGGACTCCTGGCACAT |
| MMP2 R primer | TACTCGCCATCAGCGTTCCCAT |
| MMP9 F primer | GCTGACTACGATAAGGACGGCA |
| MMP9 R primer | TAGTGGTGCAGGCAGAGTAGGA |
| GAPDH F primer | TGGCAAAGTGGAGATTGTTGCC |
| GAPDH R primer | AAGATGGTGATGGGCTTCCCG |
